# Changes in the swallowing pattern and tongue posture during the transition from deciduous to mixed dentition—a longitudinal ultrasonography study

**DOI:** 10.1093/ejo/cjad066

**Published:** 2023-11-24

**Authors:** Rok Ovsenik, Maja Marolt Mušič, Jasmina Primožič

**Affiliations:** Department of Dental and Jaw Orthopaedics, Faculty of Medicine, University of Ljubljana, Ljubljana, Slovenia; Department of Radiology, Institute of Oncology, Ljubljana, Slovenia; Department of Dental and Jaw Orthopaedics, Faculty of Medicine, University of Ljubljana, Ljubljana, Slovenia

**Keywords:** swallowing pattern, tongue posture, ultrasonography, deciduous dentition, mixed dentition

## Abstract

**Background:**

Given the importance that swallowing pattern and tongue posture might have in the aetiology of malocclusion, it appears important to be aware of the physiological changes of tongue function and its posture.

**Objectives:**

The study aimed to assess changes of the swallowing pattern and tongue posture during the transition from the deciduous to mixed dentition and the association between them.

**Materials and Methods:**

The study included 57 subjects aged 5.87 ± 0.5 with normal occlusion, orofacial functions, no history of trauma, or orthodontic treatment. Ultrasonography was used for the assessment of tongue posture and swallowing pattern, where the spontaneous act of swallowing was recorded. To evaluate the possible effect of incisors’ eruption, the swallowing pattern and tongue posture ultrasonograms were compared at the deciduous (DD), early mixed (EMD), and intermediate mixed (IMD) timepoints.

**Results:**

A significant association between the swallowing pattern and tongue posture at the DD and IMD timepoint was found. Moreover, the visceral swallowing pattern decreases with age (odds ratio [OR] = 0.777), as well as with a prolonged phase IIa (OR = 0.071), while it increases when the tongue is postured on the mouth floor (OR = 5.020).

**Limitations:**

The young age of the investigated subjects, direct contact of the probe, and the determination of the rest phase of the tongue were considered limitations.

**Conclusions:**

No statistically significant changes in swallowing pattern and tongue posture occurred during the transition period; however, a significant association between the swallowing pattern and tongue posture among subjects with normal occlusion, regardless of the dentition phase was detected.

## Introduction

The role of swallowing pattern in the aetiology of malocclusion has yet to be elucidated, despite several studies reporting a higher prevalence of malocclusion among patients with a visceral swallowing pattern [1–3]. On the contrary, others claim that a visceral swallowing pattern on its own has a negligible effect on the aetiology of malocclusion due to its relatively short duration in time of an act of swallowing [4, 5].

Some authors have therefore advocated that tongue posture, rather than swallowing, plays an important role in the aetiology of malocclusion [6–9]. It has been claimed that swallowing pattern changes during growth and development, and according to Graber *et al*., between the ages of 2 to 4 a gradual evolution from visceral to somatic pattern occurs [10]. However, a change back to a visceral swallowing pattern was reported during the transition period from deciduous to mixed dentition [11]. This is frequently regarded as normal, since during incisors’ eruption, an interincisal position of the tongue tip is considered physiologic [11, 12].

Nevertheless, a longitudinal study reported that not all children develop a somatic pattern even by the age of 12 [1]. In fact, according to Ovsenik *et al*., 25% of the examined subjects with different malocclusion preserved a visceral swallowing pattern until the permanent dentition period, which is referred to as an atypical swallowing pattern [1]. The reported study assessed the swallowing pattern during clinical examination by slightly parting the lips of the subject [13] and by palpating the contraction of the temporal muscle and the masseter muscle [14], which might have some methodological pitfalls related to the subjective assessment.

To overcome this limitation, a recent study has proposed ultrasonography of the tongue and submental area to objectively determine the swallowing pattern and its specific phases [15, 16]. More in detail, using a two-dimensional display in brightness mode (B-mode) and in real time, and later movement mode (M-mode) enabled quantitative assessment of the movement of the tongue during an act of swallowing [2]. Moreover, ultrasonography was also proposed as an objective method for tongue posture evaluation [7].

To our best knowledge, there are limited data available in the literature about the longitudinal changes of the swallowing pattern and tongue posture among subjects with normal occlusion in the pre-pubertal growth period.

Therefore, this study aimed to assess the possible changes of the swallowing pattern and tongue posture during the transition from the deciduous to mixed dentition. Further objectives were to determine whether the eruption of incisors affects the swallowing pattern and if a correlation exists between the tongue posture and its function during swallowing.

## Subjects and methods

This prospective longitudinal study, approved by the Slovenian National Medical Ethics Committee (Ref. No. 0120-127/2018-12), was performed at the Department of Orthodontics and Dentofacial Orthopaedics at the University Medical Centre Ljubljana. Before inclusion in the research, the subjects were thoroughly informed about the course of the study and written consent for participation was obtained.

Although it was calculated that a sample size of 52 subjects per group was necessary to detect an effect size coefficient of 0.4 for dependent comparisons, with an α set at 0.05 and a power of 0.80, 60 subjects were included at baseline to account for possible drop-outs. All the subjects were selected from Slovenian elementary schools and kindergartens. We included subjects with a complete deciduous dentition, flush terminal plane occlusion with the correct overjet and overbite (1–4 mm), no midline deviation, without orofacial functional impairment, and no history of trauma or previous orthodontic treatment. Subjects with a malocclusion were excluded. The final sample consisted of 57 subjects, 30 boys and 27 girls, aged 5.9 ± 0.5 due to three drop-outs at the first follow-up.

Swallowing pattern and tongue posture were evaluated longitudinally with an ultrasound (Voluson-i, GE, USA).

For the evaluation of the swallowing pattern, a spontaneous act of (empty) swallowing was considered, since it occurs most frequently, approximately once a minute in healthy young individuals [17]. The subjects sat in a chair with the head positioned on the backrest to ensure greater accuracy of the ultrasound examination by reducing head movements. They were first given 10 ml of water, in order to avoid the occurrence of dry mouth and consequently lack of saliva. After a 10-s rest period, for clear differentiation between the swallowing of water and a spontaneous swallowing, one spontaneous act of (empty) swallowing, was evaluated during the examination [7].

The evaluation was repeated multiple times when no spontaneous swallowing was detected, but instead, only ‘fake’ movement of the tongue was seen in the image. The examination was repeated until a spontaneous swallowing act was detected on the ultrasound screen.

**Figure 1.**
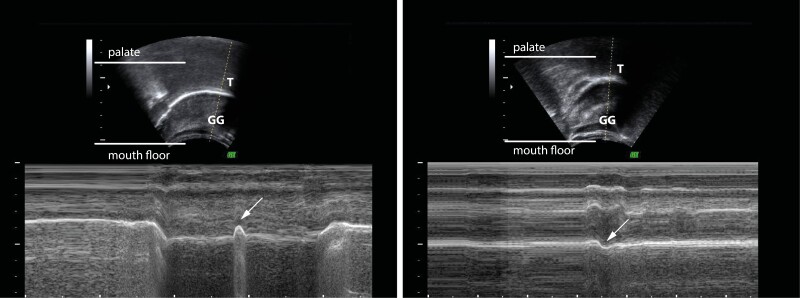
B- and M- mode tongue ultrasonogram with the ultrasound probe’s centre line set through the tip of the tongue. The white arrow shows an upward movement of the tongue tip and the genioglossus muscle in the M-mode image in Fig. 1a, which is characteristic of the somatic swallowing pattern. A downward movement of the tongue tip and the genioglossus muscle is shown by the white arrow in Fig. 1b, which is characteristic of the distinctive visceral swallowing pattern. T—tongue tip, GG—genioglossus muscle.

To determine the swallowing pattern, we observed the movement of the tongue in B—and M—modes. The centre line of the ultrasound probe was directed through either the tip and/or the genioglossus muscle or the centre of the tongue to determine the movement of both parts of the tongue. When directing the centre line of the ultrasound probe through the tip of the tongue and the more reliable genioglossus muscle [2], two types of curves occurred on the ultrasound image in M-mode. The swallowing pattern was diagnosed according to Peng *et al*., and a somatic swallowing pattern was recorded when the curve was directed upwards at the beginning of the swallowing act (Fig. 1a). In contrast, a visceral swallowing pattern was recorded when the direction of the curve was downwards [2](Fig. 1b).

We monitored the movement of the tongue during swallowing on a two-dimensional recording of the tongue, saved the recording and later analysed it with the software 4D View 17 (GE Healthcare Austria Gmbh & CoOG, Austria).

**Figure 2.**
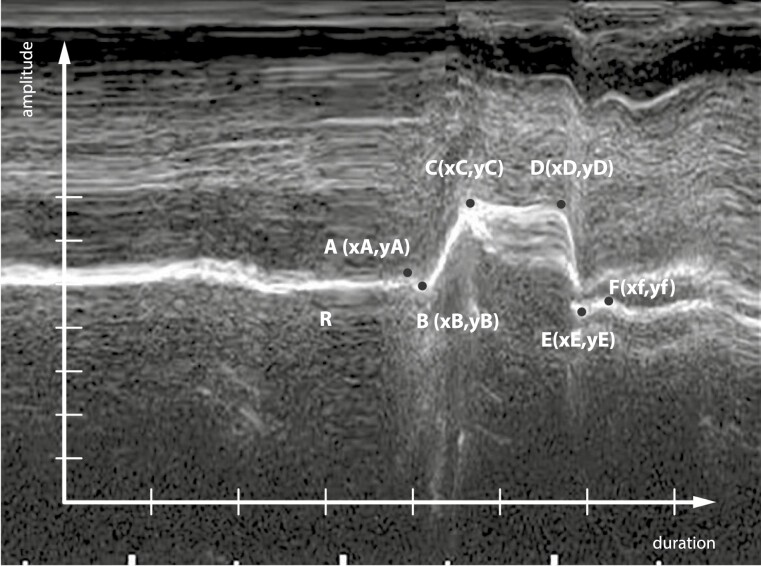
M-mode tongue ultrasonogram with the ultrasound probe’s centre line set in the middle of the tongue and presentation of swallowing phases (I, IIa, IIb, IIIa, and IIIb [15];). In the rest phase (R), the tongue tip is usually positioned on the lingual surfaces of incisors or in contact with the incisive papilla. The swallowing act starts with the shovel phase (A–B; phase I), in which the tongue tip moves cranially, and the middle third of the tongue becomes concave, reflected in the down-movement of the curve in the M-mode ultrasonogram. In the early transport phase (B–C; phase IIa), the tongue moves cranially and distally, the middle third of the tongue approaches the hard palate, and therefore the concavity disappears. The late transport phase (C–D, phase IIb) is characterised by minimal vertical movement of the tongue because of the distal transportation of saliva. In the early final phase (D–E, phase IIIa), the curve in the M-mode ultrasonogram drops because of the lowering of the mouth floor. In the late final phase (E–F; phase IIIb), the tongue returns to the rest position, reflected as a rise in the M-mode curve [15].

Based on the diagram in M-mode, the act of swallowing was divided into 5 phases: I—shovel phase; IIa—early transport phase; IIb—late transport phase; IIIa—early final phase; IIIb—late final phase [10, 18]; (Fig. 2).

Tongue posture was determined with a convex probe that automatically moves the ultrasound beam in the shape of a fan and records the selected volume of tissue. Three-dimensional reconstructions of the tongue at rest position and visualizations of it in different spatial planes were made. Tongue posture was diagnosed in a relaxed position, instructing the subject to remain still for 15 s with no movement of the tongue, in order for the ultrasound beam to have enough time collect the 3D data of the tongue posture. The evaluation was repeated multiple times, when tongue movement occurred during the examination process, resulting in an inappropriate and disturbed 3D reconstruction of the tongue, since only a resting tongue posture allows the proper 3D reconstruction of the tongue to be made.

**Figure 3.**
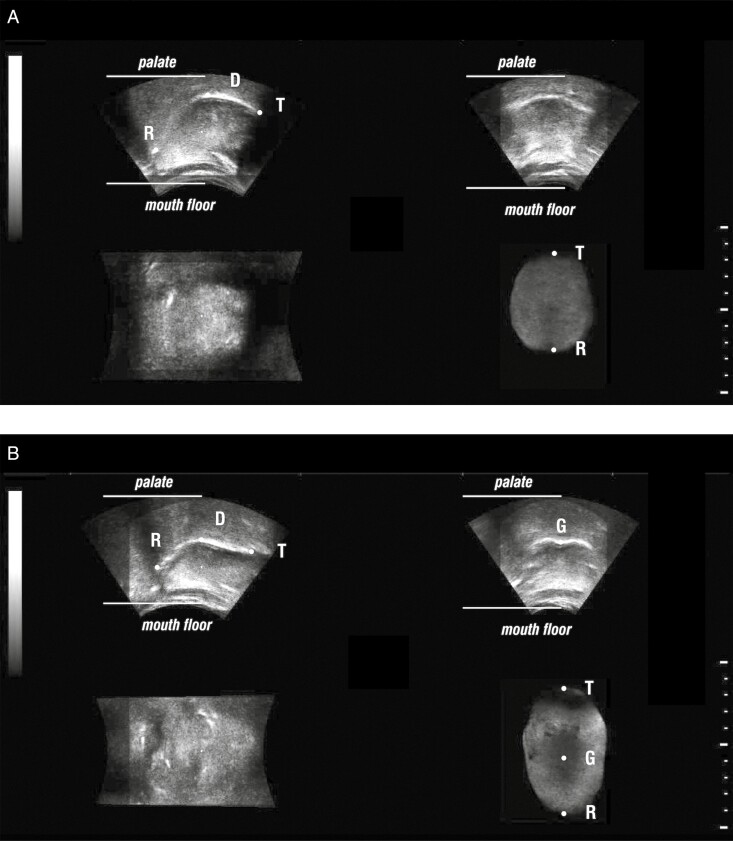
3-D ultrasonograms of tongue postured on the palate, with its convex surface and no groove in Fig. 3a, while the characteristic groove is visible in Fig. 3b, meaning that the tongue is postured on the mouth floor; R—tongue radix, D—tongue dorsum, T—tongue tip, G—groove.

According to Volk *et al*., when a characteristic groove on the back of the tongue was visible, a mouth floor tongue posture was recorded; while when the tongue’s surface was convex, a palatal tongue posture was recorded [7] (Fig. 3).

To assess the effect of the eruption of incisors, the swallowing pattern and tongue posture ultrasonograms were clustered according to dentition into the deciduous (DD), early mixed (EMD), and intermediate mixed (IMD) dentition timepoints. More in detail, the EMD recording of the swallowing pattern and tongue posture was analysed when an anterior interincisal gap was first diagnosed at the follow-up examination. In contrast, the IMD recording was analysed, when all the incisors and first permanent molars were first analysed to be fully erupted. The mean age of the included subjects at the EMD timepoint was 6.9 ± 0.8, while at the IMD timepoint, they were 8.4 ± 1.0 years old.

### Statistical analysis

After testing the normality of the data with the Shapiro-Wilk test and Q–Q normality plots of the residuals and the equality of variance among the datasets using the Levene test, non-parametric methods were used for data analysis. The Mann–Whitney *U* test was performed to test the differences in age and duration of transition from one timepoint to another among sexes. A Friedman test was used to assess differences in the duration and amplitudes of the different swallowing phases between timepoints. When significant differences were found, pair-wise comparisons were performed using the Wilcoxon test. To assess the differences in the frequency of the type of swallowing pattern and tongue posture between the timepoints, the Cochran’s *Q* test was used.

The backward multiple logistic regression analysis was used to assess any possible associations between the type of swallowing pattern (dependent variable) and the swallowing phase duration/amplitude, dentition, and tongue posture (explanatory variables). The swallowing phase duration and amplitude were entered as continues, while the type of dentition and tongue posture were entered as categorical data. Clinical and three-dimensional postural parameters (either qualitative or quantitative) were entered as categorical and continuous variables, respectively. The odds ratios (ORs) along with their 95% confidence intervals (CIs) were calculated. Sex and age were also entered in the model to adjust the ORs, and the cut-off levels of significance used were 0.05 and 0.10 for entry and removal, respectively.

The repeatability for each categorical (type of swallowing and tongue posture) parameter was calculated using the unweighted kappa coefficients, which were calculated based on 15 pairs of randomly selected assessments repeated 1 week apart. However, to quantify the full method error of the phases’ duration and amplitude measurements, the method of moments variance estimator was used. This analysis was also performed on the 15 pairs of randomly selected assessments repeated 1 week apart and was calculated for each parameter as mean and 95% CI.

The Statistical Package for Social Sciences software 20.0 (SPSS® Inc., Chicago, IL, USA) was used to perform the statistical analysis and a *P*-value of less than .05 was considered as significant.

## Results

The repeatability of the swallowing pattern and tongue posture assessments were moderate, with κ coefficient values of 0.722 and 0.595, respectively. Method errors for the specific swallowing phase duration measurements ranged from 0.04 (0.03–0.07) to 0.10 (0.08–0.17), for phase I and phase IIIa, respectively, while method errors for the specific swallowing phase amplitude measurements ranged from 0.30 (0.18–0.87) to 0.72 (0.51–1.27) for phase I and phase IIIa, respectively.

The mean age of female/male subjects at DD, EMD and IMD timepoints and the mean duration of the transition from DD to EMD and EMD to IMD timepoints for female/male subjects is presented in Table 1. There were no statistically significant differences in the mean age of female or male subjects in either DD, EMD and IMD timepoints, while the duration of transition from DD to EMD and EMD to IMD timepoint has also shown no statistically significant differences between both genders.

**Table 1.** The mean age of female/male subjects at DD, EMD, and IMD timepoints and mean duration of transition from DD to EMD and EMD to IMD timepoints for female/male subjects.

		DD	EMD	IMD
AGE (years)	Female (*N* = 27)	5.8 ± 0.5	6.7 ± 0.7	8.2 ± 0.9
	Male (*N* = 30)	5.9 ± 0.5	7.0 ± 0.7	8.5 ± 0.8
	*P* value	0.16	0.08	0.22

		DD-EMD		EMD-IMD
DURATION (months)	Female (*N* = 27)	11.3 ± 5.5		17.6 ± 7.8
	Male (*N* = 30)	13.0 ± 5.5		17.4 ± 6.9
	*P* value	0.14		0.82

The percentage of somatic/visceral swallowing patterns and tongue posture on the palate/mouth floor is presented in Table 2. The highest rate of somatic swallowing pattern was present at the IMD timepoint. In contrast, the highest percentage of tongue posture on the palate was present at the EMD timepoint, however, with no statistically significant differences between the three timepoints.

**Table 2.** The prevalence of either somatic or visceral swallowing pattern and tongue posture on the palate or mouth floor at the DD, EMD, and IMD timepoint.

Parameter	Timepoint			*P* value
	DD	EMD	IMD	
Swallowing pattern (somatic/ visceral; %)	45.6/54.4	42.1/ 57.9	54.4/ 45.6	.368
Tongue posture (palate/ mouth floor; %)	57.9/42.1	61.4/38.6	59.6/40.4	.918

**Figure 4.**
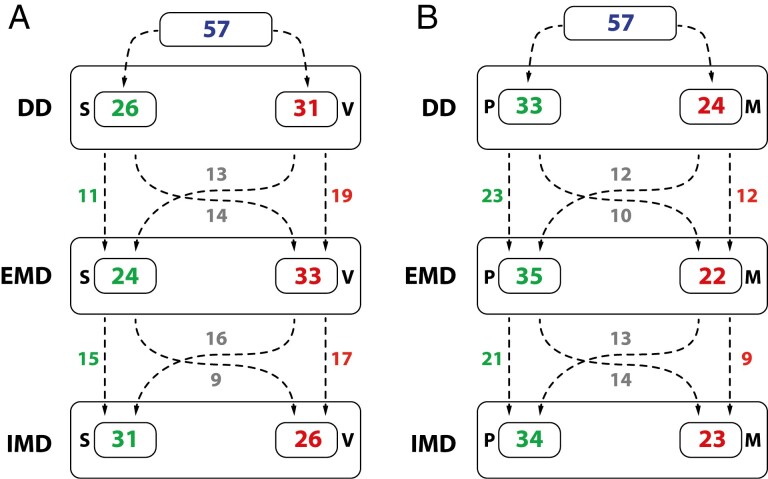
The number of subjects with a specific swallowing pattern (a) and tongue posture (b) and the number of subjects with a change/ no change of the swallowing pattern (a) and tongue posture (b) during the transition from DD, EMD and IMD timepoints; S—somatic swallowing pattern, V—visceral swallowing pattern, P—tongue postured on the palate, M—tongue postured on the mouth floor.

The frequency of subjects with a specific swallowing pattern and tongue posture according to the specific dentition timepoint is shown in Fig. 4. Of all subjects, 31.6% had a stable swallowing pattern through all the timepoints, meaning no change occurred. However, the swallowing pattern changed during the three timepoints once in 45.6% of subjects, while it changed twice in 22.8% of subjects (Fig. 4a).

Tongue posture (Fig. 4b) has not changed between the timepoints in 36.8% of subjects, resulting in a stable position. However, 38.6% and 24.6% of subjects had one and two changes in tongue posture during the timepoints, respectively.

Table 3 represents the mean duration and amplitude of each swallowing phase at different timepoints, with no statistically significant differences between all three timepoints. The only exception was detected for the amplitude of phase IIIa, however, at pair-wise comparisons, no significant differences were detected.

**Table 3.** Mean duration and amplitude of each swallowing phase at DD, EMD, and IMD timepoints.

Parameter		Timepoint			*P* value
		DD	EMD	IMD	
	Phase I	0.123	0.154	0.150	.642
	Phase IIa	0.260	0.294	0.228	.069
	Phase IIb	0.980	0.108	0.131	.629
	Phase IIIa	0.152	0.147	0.143	.659
Duration (s)	Phase IIIb	0.151	0.132	0.127	.404
	Phase I	0.213	0.211	0.222	.995
	Phase IIa	0.794	0.847	0.744	.794
	Phase IIIa	0.924	1.03	1.025	.044
Amplitude (mm)	Phase IIIb	0.214	0.176	0.151	.115

When looking at the possible correlation between swallowing pattern and tongue posture at each of the dentition timepoints, we found that there is a significant correlation between swallowing pattern and tongue posture at the DD (*P* < .001) and IMD timepoint (*P* = .003). In contrast, no correlation was shown at EMD timepoint.

The logistic regression analysis results (Table 4) evidenced a decrease in the risk of visceral swallowing patterns with age. On the contrary, tongue posture on the mouth floor increases the risk of visceral swallowing pattern by five times. Furthermore, when phase IIa is prolonged by 1 s, it decreases the risk of visceral swallowing.

**Table 4.** Results of the logistic regression analysis with age, tongue posture and duration of phase IIa as independent variables and swallowing pattern as the dependant variable.

	B	SE	*P* value	OR	Lower 95% CI	Upper 95% CI
Age	−0.253	0.131	.054	0.777	0.601	1.004
Tongue posture	1.614	0.354	.000	5.020	2.509	10.047
Duration of phase IIa	−2.648	1.217	.030	0.071	0.007	0.769

R^2^ refers to the whole regression model.

B, unstandardized regression weight; SE, multiple linear regression/ standard deviation; *P*-value, determinant of the significance of the variables; OR, odds ratio; CI, confidence interval for OR.

## Discussion

The present study showed a similar prevalence of somatic and visceral swallowing patterns at DD, EMD, and IMD timepoints. Moreover, the percentage of subjects with visceral swallowing pattern increased from DD to EMD timepoint and then decreased from EMD to IMD timepoint, but the change was not statistically significant. Given the importance that swallowing pattern, but even more tongue posture, might have in the aetiology of malocclusions, it appears important to be aware of the physiological changes of tongue function and posture in subjects with no malocclusions, especially during the sensitive transitory period of growth and development in the orofacial area. Graber believed that swallowing pattern should mature into somatic by the age of four [10]. On the contrary, the present study evidenced a prevalence of 54.4% of persisting visceral swallowing pattern by the age of 6. Interestingly, at the EMD timepoint, this prevalence further increased to 57.9%; however, no significant differences were observed between the two timepoints. This increase could be explained by the interposition of the tongue between the erupting incisors as an adaptation to the occurrence of a transitory anterior gap, where the tongue acts as a seal. At the IMD timepoint, the percentage of subjects with a visceral swallowing pattern insignificantly decreased. Nevertheless, 45.6% of subjects had a persisting visceral swallowing pattern. Similar findings were reported by Melsen *et al*. [14], confirming that visceral swallowing pattern remains present in 25–30% of 9-year olds, and Ovsenik *et al*. [1] reporting the persistence of visceral swallowing pattern in 35% of the 8-year olds.

Besides no statistically significant differences in the prevalence of different swallowing pattern and tongue posture between the three timepoints, no significant differences in the amplitudes and durations of each swallowing phase were observed as well. On the contrary to our findings, Volk *et al*. reported a prolonged late transport phase, along with a prolonged act of swallowing as a whole. At the same time, the amplitude of the tongue movement during a swallowing act in the same investigated group was also reported to be larger [16]. Also on the contrary to our study, Peng *et al*. reported a prolonged late transport phase in subjects with a visceral swallowing pattern [19].

In the present study, a prolonged early transport phase decreased the possibility of the occurrence of visceral swallowing pattern in individuals, which is in contrast to the reported results of Peng *et al*. [2], who have stated that apparent differences in tongue movement between the visceral and the somatic groups could only be identified in the shovel phase of a swallowing act, since according to Shawker *et al*., tongue movement in all the other four phases was considered reflex [20]. One of the possible explanations for the difference in reported results could be that their investigated group was in the late mixed to permanent dentition phase, with the lowest average age being 10 years. At the same time, our subjects were younger, as they were being monitored from 5 to 8 years of age. Subjects with malocclusions or with involvement in orthodontic treatment have been excluded in both studies. In visceral swallowing pattern, the tongue has a shorter path to travel in this phase, since it stretches and inserts itself between the upper and lower teeth [19], while it has to achieve greater distance in somatic swallowing pattern, until contacting the portion of the hard palate behind the upper incisors. This could be the reason why a prolonged early transport phase would lead to a decreased possibility for the occurrence of visceral swallowing pattern.

Besides the correlation between a prolonged early transport phase and swallowing pattern (OR = 0.071, *P* < .05), we also found that the risk of visceral swallowing decreases with age (OR = 0.777, *P* < .05). In their study, Ovsenik *et al*. also reported the decreasing prevalence of visceral swallowing pattern from the ages of 6 to 9 and even 12 [1]. On top of that, it was shown in our study that tongue posture on the mouth floor significantly increases the risk of visceral swallowing pattern by five times (OR = 5.020; *P* < .01). This detailed association and its exact effect have not yet been described in the literature in such manner. It has, however, similarly already been stated that open mouth with the accompanying tongue posture on the mouth floor is one of the most important disturbances leading to multiple orofacial function impairment, with swallowing, as a function, being significantly affected [9, 14]. Furthermore, Fujiki *et al*., using cineradiography, suggested that protrusive movement of the tongue during swallowing could be a consequence of the downward and forward positioning of the tongue [21, 22]. This would be in accordance with the results of our study.

The present study also showed a similar prevalence of tongue posture on either the palate or the mouth floor at DD, EMD, and IMD timepoints. The results have shown that the prevalence of tongue postured on the mouth floor decreases from DD to EMD, but then increases again in IMD, with the change not being statistically significant. Proffit believed that tongue posture plays a much more critical role in the aetiology of malocclusion than its function [4]. The present study evidenced a prevalence of 42.1% of tongue postured on the mouth floor at the DD timepoint. Interestingly, the prevalence of subjects with tongue postured on the mouth floor decreases from DD to EMD timepoint to 38.6%, but then increases again to 40.4% at IMD timepoint, however, with no statistically significant differences between the three timepoints. The contradicting finding between the swallowing pattern and tongue posture at the EMD timepoint may suggest that the occurrence of a transitory anterior gap between the erupting incisors has more effect on the function of the tongue. In contrast, tongue posture at its spontaneous resting position remains stable during the transition. Similar findings were reported in an epidemiologic study by Stahl and Grabowski [23], where a slightly lower (27%) prevalence of tongue posture dysfunction was present in the DD, while no statistically significant change from DD to EMD was noted, following our present study.

When looking at the possible association between swallowing pattern and tongue posture in each dentition, we found a significant correlation between swallowing pattern and tongue posture in the deciduous and intermediate mixed dentition. This is in accordance with the studies from Volk *et al*. and Ovsenik *et al*., where the authors emphasised the role of both orofacial functions, swallowing as well as tongue posture, as important factors in the early aetiology of unilateral posterior crossbite, since the majority of children with unilateral posterior crossbite had tongue postured on the mouth floor [7], while visceral swallowing pattern was found in 83% of the children with this malocclusion [16]. Furthermore, a recent study [24] stated that an altered tongue position during its function leads to an imbalance between oral tissue forces and thus may produce changes in skeletal and dentoalveolar tissue, which has already been emphasised in the functional matrix theory by Moss [25].

### Limitations

The young age of the included subjects might be considered a limitation, related to compliance and thus resulting in a moderate repeatability of the results, which by itself is a limitation of the method. However, Volk *et al*. [7], also using ultrasonography in children of similar age, concluded that the swallowing pattern and tongue posture could be reliably assessed in such a young population. Nevertheless, the repeatability of the assessments in the present study was moderate. Another limitation was the determination of the rest phase of the tongue in a swallowing act, since very often, there is a difference in the tongue’s position at the beginning and the end of one swallowing act. Furthermore, some individuals do not swallow only once but rather make multiple acts of swallowing sequentially, making it hard to pinpoint the exact moment when the tongue is resting. To overcome this limitation, we used the principle of Cheng *et al*. [18], where the tongue’s rest position was identified as soon as the tongue surface and muscles on the M-mode image came to a flat signal, while also taking ultrasonograms of a spontaneous swallowing act, rather than an indicated one. Since the ultrasound probe is in direct contact during the examination of a child to determine both tongue posture and swallowing pattern, it could act as a disturbing factor for a child to act spontaneously during the examination, thus altering the posture of the tongue and the way of swallowing. To decrease the limitation as much as possible, the ultrasound examination was repeated multiple times to correctly assess the spontaneous swallowing pattern as well as the resting posture of the tongue [26].

## Conclusions

The present study showed that almost half of the subjects with normal occlusion still had a persisting visceral swallowing pattern and tongue postured on the mouth floor until as late as the IMD. The function of the tongue seems more affected by the anterior gap occurring during the incisors’ eruption, while the tongue posture remains relatively stable throughout the transition from the deciduous to the permanent dentition in the intercanine part of the dental arch. Moreover, the visceral swallowing pattern is inversely correlated with children’s age, while tongue postured on the mouth floor and the prolonged early transport phase of the swallowing act are both positively significantly correlated with the visceral swallowing pattern among subjects with normal occlusion.

## Data Availability

The data supporting this study’s findings are available on request from the corresponding author, R.O. The data are not publicly available due to their containing information that could compromise the privacy of research participants.
